# No Effect of Lactobacillus rhamnosus GG on Eradication of Colonization by Vancomycin-Resistant Enterococcus faecium or Microbiome Diversity in Hospitalized Adult Patients

**DOI:** 10.1128/spectrum.02348-21

**Published:** 2022-04-27

**Authors:** Ingrid Maria Cecilia Rubin, Sarah Mollerup, Christa Broholm, Signe Boye Knudsen, Adam Baker, Morten Helms, Mona Katrine Alberthe Holm, Thomas Kallemose, Henrik Westh, Jenny Dahl Knudsen, Mette Pinholt, Andreas Munk Petersen

**Affiliations:** a Department of Clinical Microbiology, Copenhagen University Hospital - Amager and Hvidovre, Hvidovre, Denmark; b Department of Gastroenterology, Copenhagen University Hospital - Amager and Hvidovre, Hvidovre, Denmark; c Chr. Hansen A/S, Human Health, Hoersholm, Denmark; d Department of Clinical Microbiology, Copenhagen University Hospital, Herlev, Denmark; e Department of Infectious Diseases, Copenhagen University Hospital - Amager and Hvidovre, Hvidovre, Denmark; f Clinical Research Centre, Copenhagen University Hospital - Amager and Hvidovre, Copenhagen, Denmark; g Institute of Clinical Medicine, University of Copenhagen, Copenhagen, Denmark; h Department of Clinical Microbiology, Copenhagen University Hospital Rigshospitalet, Copenhagen, Denmark; University of Parma

**Keywords:** vancomycin-resistant *Enterococcus faecium*, gut microbiome, *Lactobacillus rhamnosus*, probiotics

## Abstract

The purpose of this trial was to evaluate the efficacy of a 4-week supplementation of Lactobacillus rhamnosus GG (LGG) in eliminating the gastrointestinal carrier state of vancomycin-resistant Enterococcus faecium (VREfm) in hospitalized adults. The primary outcome of the study was the number of patients with cleared VREfm colonization after the 4-week intervention. Secondary outcomes were clearance of VREfm colonization at weeks 8, 16, and 24, number of VREfm infections (isolated from nonintestinal foci), and changes in fecal microbiome diversity after the intervention. The trial was a multicenter, randomized, double-blind, placebo-controlled trial in hospitalized adult VREfm carriers. Patients were enrolled and randomized to receive 60 billion CFU of LGG daily or placebo for 4 weeks. For a subgroup of patients, rectal swabs for VREfm were collected also at 8, 16, and 24 weeks and analyzed using shotgun metagenomics. Patients ingesting a minimum of 50% of the probiotic during the 4-week intervention were included in subsequent outcome analyses (48 of 81 patients). Twelve of 21 patients in the LGG group (57%) compared to 15 of 27 patients in the placebo group (56%) cleared their VREfm carriage. Eighteen patients completed the entire 24-week intervention with the same minimum compliancy. Of these, almost 90% in both groups cleared their VREfm carriage. We found a statistically significant difference between VREfm clearers and nonclearers regarding metronidazole and vancomycin usage as well as length of hospitalization after inclusion. The microbiome analyses revealed no significant difference in alpha diversity between the LGG and the placebo group. Beta diversity differed between the groups and the different time points. This study did not show an effect of LGG in eradication of VREfm after a 4-week intervention.

**IMPORTANCE** Whereas other studies exploring the effect of L. rhamnosus in clearing VREfm from the intestine included children and adults, with a wider age range, our study consisted of a geriatric patient cohort. The natural clearance of VREfm in this study was almost 60% after 4 weeks, thus much higher than described previously. Also, this study characterizes the microbiome of VREfm patients in detail. This article showed no effect of the probiotic L. rhamnosus in clearing VREfm from the intestine of patients.

## INTRODUCTION

Enterococcus faecium is a commensal of the gut, with the potential for causing a variety of infections, such as urinary tract infections, bacteremia, intraabdominal infections, and indwelling catheter-related infections (1, 2). In Denmark, the preferred antibiotic to treat invasive E. faecium infections is vancomycin, and therefore the increase in vancomycin-resistant E. faecium (VREfm) since 2013 has been a concern for the Danish health authorities (3, 4). VREfm is endemic in hospital settings, and infections are associated with longer hospitalization and higher mortality than those with vancomycin-sensitive enterococci (5, 6). In the United States, VREfm caused an estimated 54,500 infections among hospitalized patients in 2017 with an annual attributable health care cost of 539 million US dollars (7). As this bacterium has an ability to develop resistance to most antibiotics used for treatment, novel approaches are envisaged to lower the development rate of antibiotic resistance (8). Vancomycin-resistant enterococci are now widespread nosocomial pathogens, where asymptomatic, colonized patients can be a source of transmission and patient-to-patient transmission is the main route by which VREfm is spread (9). Risk factors for contracting VREfm are prolonged hospitalization, close proximity to carrier patients, immunosuppression, comorbidities, and use of antibiotics, especially vancomycin (8, 10, 11). Colonization with VREfm can lead to clinical VREfm infections, especially in patients who are hospitalized for a long time, patients with indwelling catheters, patients treated with vancomycin, and immunocompromised patients (7, 12–14). It is, therefore, important to lessen the burden of VREfm colonization. Currently, antibiotic stewardship and infection control precautions are the most important measures to prevent transmission, as there is no treatment for VREfm colonization (15).

The gut microbiome plays an important role in preventing colonization by pathogenic bacteria, such as VREfm (16, 17). In patients who receive broad-spectrum antibiotics, the microbiome is disrupted, posing a risk for acquisition and colonization by multidrug-resistant (MDR) bacteria, such as VREfm (8). Treatment with vancomycin is shown to have detrimental long-term effects on the human gut microbiome (18). It is thought that VREfm outbreaks in Denmark were partly due to the increased use of vancomycin, as it coincided with a large outbreak of Clostridioides difficile infections (CDI) and increased use of vancomycin at around the same time (19). Many studies have shown a large impact by both hospitalization and antibiotic use (16, 20). In one study, only 21% of the gut microbiota at admission remained constant after 24 h of resampling, measured by the mean Jaccard distance over time, where the Jaccard distance was used to measure dissimilarity between gut communities (21).

To reverse gut dysbiosis and restore a healthy microbiome, probiotics can be used. *Lactobacillus*, a genus of lactic acid-producing, Gram-positive bacteria, and a well-documented probiotic, has been used in food and dietary supplements since 1990 (22). Lactobacillus rhamnosus GG (LGG) might prevent dysbiosis of the gut, thus restoring the healthy microbiome and thereby preventing colonization by pathogenic bacteria. Various mechanisms have been proposed for the effects of lactobacilli, including inhibition of growth of pathogens or a direct bactericidal effect exerted by secreted molecules, inhibition of expression of virulence genes, outcompetition of pathogenic bacteria by competition for binding sites, and stimulation of antimicrobial host intestinal cell responses (23, 24). In an experimental model, LGG was also shown to directly outcompete VREfm (25).

Previous studies have investigated the potential of LGG to reduce the gastrointestinal carriage of VREfm in different patient populations with various results and, generally, with a lack of study power (26–28). In this study, we sought to investigate whether a 4-week intervention with LGG could increase the numbers of VREfm clearers in hospitalized adult patients, as well as possible changes in the diversity of the microbiome.

## RESULTS

**FIG 1:**
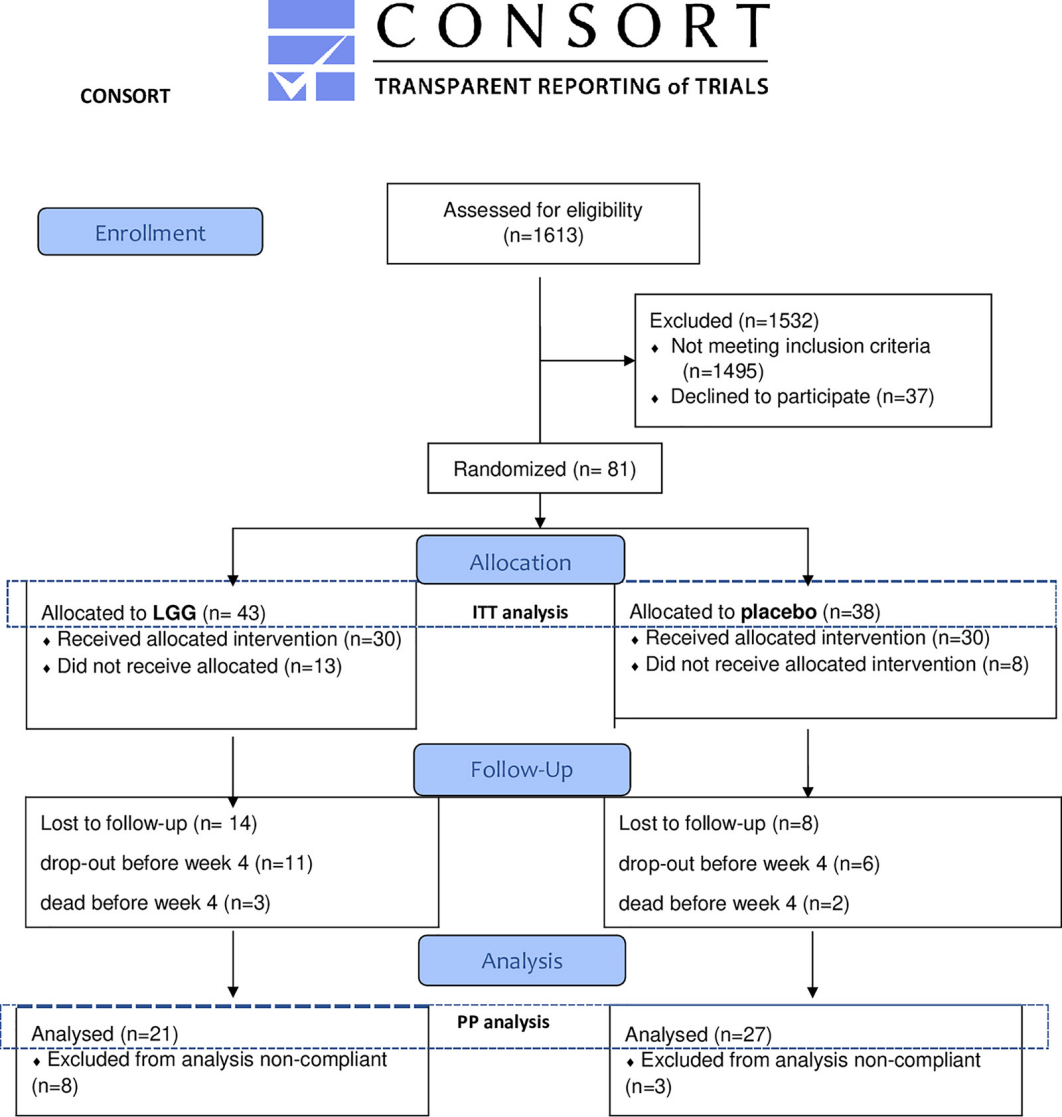
Flowchart based on CONSORT Reference (48) for patients screened and enrolled in the study. ITT, intention to treat; PP analysis, per protocol analysis.

In this study, 1,613 VREfm-positive patients were identified and screened for eligibility, with 116 patients satisfying the inclusion criteria. Thirty-seven patients declined to participate, leaving a total of 81 study participants (5%) enrolled and randomized to receive LGG or placebo twice daily (Fig. 1). The study was terminated before reaching the planned number of 162 patients, partly due to the unforeseen slow pace in patient recruiting and partly due to the COVID-19 pandemic, which almost terminated VREfm outbreaks in our hospitals, further decreasing the inclusion rate (29).

Of the 81 included participants, 48 were compliant with the study protocol and included in outcome analyses. The dropout rate was 41% (33 patients), 22 from the LGG arm and 11 from the placebo arm (*P = *0.07, nonsignificant). Most patients that dropped out were noncompliant with the study treatment or nonadherent to the study protocol. None of these participants reported adverse effects (AEs) or severe adverse effects (SAEs) related to the probiotic intervention. Three participants in the LGG group versus two in the placebo group died before week 4. None of these deaths were associated with intake of LGG (Fig. 1). AEs occurred with the same frequency in both treatment arms, and we did not find any that could be related to the ingestion of LGG. No infections with LGG occurred in either group, nor did any VREfm infections from nonintestinal foci. All SAEs occurring were reported to the Institutional Review Board, and no safety concerns were raised, nor were there any recommendations to discontinue the study drug or terminate the study.

Of the patients included in outcome analyses, 21 were in the LGG group and 27 were in the placebo group. There was no difference between the groups regarding age, gender, Charlson comorbidity score, or antibiotic use prior to inclusion (Table 1). The mean compliancy for both groups was 86%.

**TABLE 1:** Baseline demographics for all participants included in the outcome analyses

Variable	Value for group:		OR	*P* value
	LGG	Placebo		
Age, median yrs (range)	76 (71–82)	74 (64.5–82.5)		0.33
Gender, female *n* (%)	14 (67)	16 (59)	0.73 (0.22–2.39)	0.77
CMI^*a*^, median, (range)	4 (3–5)	4 (2.5–5)		0.14
Antibiotics received 6 mo prior to inclusion				
J01CE, J01A, beta-lactamase-sensitive penicillin and extended-spectrum penicillin	11	14	1.0 (0.3–3.2)	1.00
J01CF, beta-lactamase-resistant penicillin	4	9	0.5 (0.1–1.8)	0.34
J01CR, combination of penicillin, incl. beta-lactamase inhibitors	17	16	2.9 (0.8–11.1)	0.13
J01DC, J01DD, cephalosporins, 2nd and 3rd generation	4	8	0.6 (0.1–2.2)	0.51
J01DH, carbapenems	2	1	2.7 (0.2–32.4)	0.57
J01EE, sulfonamide and trimethoprim	0	1		1.00
J01FA, macrolides	5	2	3.9 (0.7–22.6)	0.21
J01FF, lincosamides	1	0		0.44
J01GB, other aminoglycosides	0	1		1.00
J01MA, fluoroquinolones	7	8	1.2 (0.3–4.1)	1.00
J01XA, glycopeptides	3	1	4.3 (0.4–45.1)	0.31
J01XD, imidazole derivates	4	4	1.4 (0.3–6.2)	0.72
J01XX, other antibacterial	4	1	6.1 (0.6–59.5)	0.15
J01AA, tetracyclines	0	0		1.00
J04AB, riphamycin	1	0		0.44
Other antibacterial	4	1	6.1 (0.6–59.5)	0.15

a CMI, Charlson comorbidity index.

### Primary outcome.

This study did not find a difference in the elimination of VREfm carriage between the groups receiving LGG or placebo after the 4-week intervention (odds ratio [OR] of 1.1, confidence interval [CI] of 0.3 to 3.7). In the LGG group, 12 of 21 study participants (57%) cleared their VREfm, whereas 15 of 27 participants in the placebo group cleared their VREfm after 4 weeks (56%) (Table 2).

**TABLE 2:** Number of participants and VREfm status in LGG group and placebo group after 4, 8, 16, and 24 weeks

VREfm status	No. of VREfm (+) and VREfm (−) patients in LGG and placebo groups at wk:				
	0 (baseline) (*n* = 48)	4 (*n *= 48)	8 (*n* = 17)	16 (*n* = 15)	24 (*n* = 18)
LGG group					
Negative	0 (0)	12 (57)	6 (75)	8 (80)	7 (88)
Positive	21 (100)	9 (43)	2 (25)	2 (20)	1 (12)
Placebo group					
Negative	0 (0)	15 (56)	6 (67)	4 (80)	9 (90)
Positive	27 (100)	12 (44)	3 (33)	1 (20)	1 (10)
					
OR (95% CI)		1.1 (0.3–3.7)	1.5 (0.2–12.5)	1 (0.1–14.6)	0.8 (0.04–14.7)
Statistical difference in VREfm loss between groups		0.912	1	1	1

### Secondary outcomes.

For the subgroup of study participants in the follow-up visits, no significant differences in VREfm clearance were seen between the groups at weeks 8, 16, and 24 (Table 2). At 24 weeks, almost 90% of patients tested in both groups had cleared their VREfm carriage.

Of interest, there was a significant difference in length of hospitalization after inclusion between clearers and nonclearers of VREfm at 4 weeks (*P = *0.026), as well as a statistically higher number of patients treated with either vancomycin (*P = *0.031) or metronidazole (*P = *0.015) among the nonclearers, although the numbers are small (Table 3).

**TABLE 3:** Analysis of the 48 patients included in outcome analyses^*a*^

Variable	Value for:		*P* value
	Clearers	Nonclearers	
Hospitalization (median days and range)			
Before	6 (0–24)	6 (0–26)	0.624
After	2 (0–16)	2 (0–44)	0.026
Treatment with antibiotics (no. of patients receiving treatment)			
Metronidazole, J01XD	1	7	0.015
Vancomycin, J01XA	0	4	0.031
Cefuroxime, J01DC	4	8	0.095
Piperacillin-tazobactam, J01CR	12	16	0.055

a The variables are hospitalization in days and treatment with antibiotics known to drive VREfm acquisition. Risk factors associated with VREfm acquisition were analyzed for number of patients clearing VREfm versus nonclearers.

### Fecal microbiome analyses.

A total of 10 of 21 patients in the LGG group and 11 of 27 patients in the placebo group were included in the microbiome analyses. Sequencing of the fecal samples resulted in a median of 2,272,816 read pairs per sample (range 533,797 to 4,637,953). After depletion of human reads, a median of >99% of reads were retained, with only two samples having <92% nonhuman reads. For the positive control, 5,153,091 read pairs were obtained, and for the negative, only 9,775 read pairs were obtained. The composition of the microbiome in terms of relative abundance (supplemental material) was presented at the family level at each sampling time (Fig. S1). An overall shift from *Enterococcus* being the dominant taxon at baseline to a domination of *Lachnospiraceae* and *Peptoniphilaceae* after 4 and 24 weeks was generally observed. E. faecium was the dominating species at baseline in several patients, and it was also higher among VREfm clearers than among nonclearers (Fig. S2). We analyzed the 10 most abundant genera at baseline and at week 4 in the LGG and placebo groups, as well as in clearers and nonclearers (Fig. S3). Overall, the same trends were observed for most of these genera, although a notable increase in *Lactobacillus* was seen for the LGG group. *Enterococcus* decreased in all groups, whereas *Blautia* and *Finegoldia* increased in all groups. *Blautia* has been described as one of the dominating genera of the microbiota, with probiotic properties (30). *Finegoldia* is also described as a part of the normal gut microbiota but can also be an opportunistic pathogen (31). Comparing clearers and nonclearers, a more noticeable decrease in *Enterococcus* was observed for the clearers. The genera *Escherichia* and *Corynebacterium* decreased in clearers but increased in nonclearers, whereas the opposite was seen for *Bifidobacterium*.

**FIG 2:**
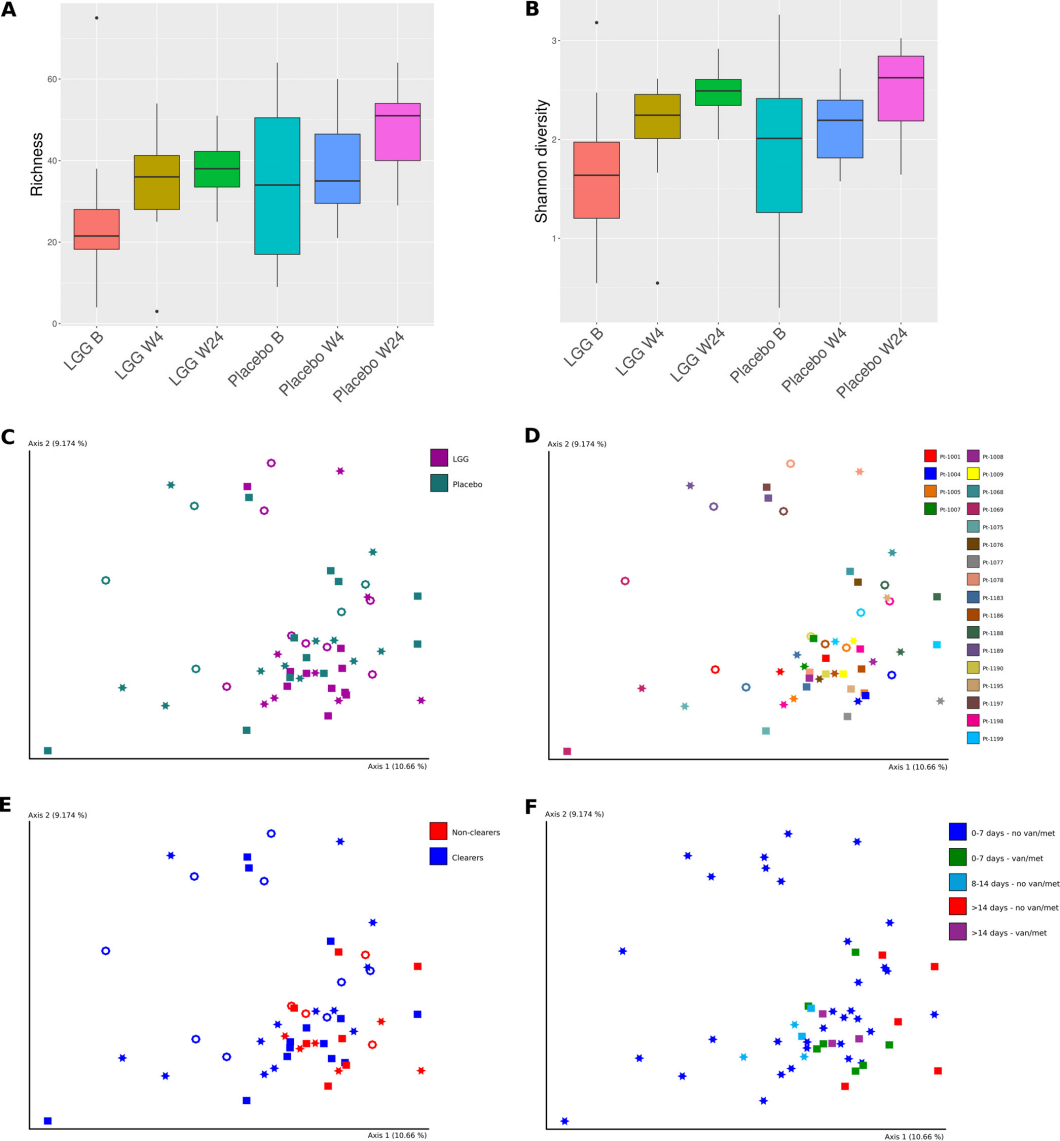
Microbiome diversity. Alpha diversity in terms of (A) richness and (B) Shannon diversity for the LGG and placebo groups at baseline, 4 weeks, and 24 weeks. Beta diversity in terms of PCoA of Aitchison distances colored by treatment arm (C), patient (D), clearers and nonclearers of VREfm (E), and length of hospitalization after inclusion as well as use of vancomycin or metronidazole (F). (C to E) Squares represent baseline, stars week 4, and open circles week 24. (F) Stars represent VREfm clearers, squares represent VREfm nonclearers. B, baseline; W4, week 4; W24, week 24; van, vancomycin; met, metronidazole.

Regarding alpha diversity, we saw a trend toward increased species richness and Shannon diversity over time for both groups (Fig. 2a), although this was not statistically significant (LGG week 4 versus baseline [richness *P = *0.195, Shannon *P = *0.25]; LGG week 24 versus baseline [richness *P = *0.148, Shannon *P = *0.0781]; placebo week 4 versus baseline [richness *P = *0.475, Shannon *P = *0.24]; placebo week 24 versus baseline [richness *P = *0.713, Shannon *P = *0.813]). The average richness and Shannon diversity for the placebo group were larger than those for the LGG group at baseline, although this was also not statistically significant (richness *P = *0.342, Shannon *P = *0.512). The same held true for VREfm clearers versus nonclearers (Fig. S4). Beta diversity analyses showed grouping by study group (LGG versus placebo at baseline, 4 weeks, and 24 weeks) and time (*P = *0.006, permutational multivariate analysis of variance [PERMANOVA], 999 permutations), by subject (*P = *0.001, PERMANOVA, 999 permutations), and by clearance of VREfm by week 4 (*P = *0.004, PERMANOVA, 999 permutations) (Fig. 2). Samples taken at baseline (squares in Fig. 2c to e) and samples from subjects not clearing VREfm (red points in Fig. 2f) were localized mainly or only to the lower right quadrant. Samples from subjects not clearing VREfm by week 4 (squares in Fig. 2f) had, with one exception, either taken metronidazole and/or vancomycin or been hospitalized for longer than 7 days after baseline, reflecting the effect of these factors on clearance of VREfm reported above.

As the relative abundances of species or genera are interdependent (32), we used ALDEx2 to identify species that were differentially abundant. ADLEx2 identified 10 species that were differentially abundant based on unadjusted *P* values (Table S1), but when considering the adjusted *P* values or the effect size, only L. rhamnosus was differentially abundant, and only for the LGG group at week 4 (*P *= 0.2 and effect size of 1.6 versus LGG baseline; *P *= 0.02 and effect size of 1.6 versus placebo week 4). For the placebo group, L. rhamnosus was detected in 1 of 11 patients at baseline (9%) but in no patients at week 4 or 24. For the LGG group, L. rhamnosus was detected in 2 of 10 patients at baseline (20%), 7 of 8 patients at 4 weeks (88%), and 1 of 8 patients at 24 weeks (13%), corresponding well with L. rhamnosus being identified as differentially abundant at week 4 in the LGG group.

The positive control from ZymoBIOMICS run in parallel with the samples includes eight bacterial species (https://www.zymoresearch.com/collections/zymobiomics-microbial-community-standards/products/zymobiomics-microbial-community-standard). Of these, Pseudomonas aeruginosa was not detected, Escherichia coli was detected at a relative abundance of 0.002 times that expected, and Salmonella enterica was detected at a relative abundance of 0.001 times that expected. Furthermore, Bacillus subtilis was misclassified as Bacillus intestinalis (Table S2).

## DISCUSSION

Our study did not find an effect of eradication of VREfm colonization by the probiotic LGG. However, our assumption that only 25% in the placebo group would clear VREfm by the end of the intervention proved to be wrong. In both groups, almost 60% of the patients cleared their VREfm after 4 weeks, and by the end of the 20-week follow-up, almost 90% had cleared their VREfm in both groups. Thus, the spontaneous clearance of VREfm was much higher than anticipated. Previous studies have shown a spontaneous clearance of VREfm of 33% during a 3-year study period (33), while a systematic review, using a logistic regression model, showed that 50% of subjects had cleared colonization 25 weeks after initial colonization (34). One aspect worth investigating further is clearance among different patient cohorts. Some patients, for example hematological patients, are both at increased risk of obtaining VREfm colonization and at increased risk of developing a VREfm infection once colonized (35). In our catchment area, we do not have hematological or oncological patients, nor did we include patients from the intensive care unit (ICU) in our study. The studies above included mainly oncological patients. This might, in part, explain the higher natural clearance of our patient cohort.

Several studies have tried to eliminate VREfm colonization using probiotics, specifically LGG. The probiotics are hypothesized to be able to outcompete VREfm, thus decreasing VREfm colonization burden and thereby the transmission between patients (28, 36). Three previous placebo-controlled randomized controlled trials have examined the effect of LGG on VREfm colonization. Szachta et al. examined the effect of LGG on 65 children with VREfm intestinal carriage and found that VREfm was temporarily eliminated from the intestine of the treatment group, with a significant effect after 3 weeks of intervention; however, the effect did not persist at week 4 (28). Manley et al. showed a sustained decrease of VREfm colonization in their study of 27 VREfm-carrying adult patients at a renal ward (26). Patients in the treatment arm received 100 g of LGG yogurt for 4 weeks, and all 11 patients in the LGG arm cleared VREfm, whereas only 1 of 12 patients in the placebo group had cleared their VREfm (*P* < 0.001). At the 8-week follow-up, eight patients remained VREfm negative, while three patients who had received antibiotic treatment were again VREfm positive in the LGG arm. The authors concluded that LGG can be used to eliminate VREfm but that larger studies are warranted. In contrast, a 2-week intervention with 20 billion CFU of LGG daily did not affect VREfm colonization, which could be explained by the shorter intervention and low LGG dosage (27).

In our study, VREfm-colonized patients were primarily elderly and comorbid, as seen by the high Charlson comorbidity index. The main exclusion criterion was inability to sign informed consent, reflecting that dementia was a characteristic of this group, and as most of our VREfm outbreaks take place at the geriatric wards, this was not surprising. Also, the fact that 40% of the included patients were noncompliant with the protocol reflected that this was a challenging patient cohort. VREfm colonization is asymptomatic, and thus not noticed by the patient, which could possibly explain the low adherence to the study treatment. The fact that more patients either dropped out or were noncompliant in the LGG arm seems, to the best of our knowledge, to be random, as there were no AEs and SAEs related to LGG in either group and, consequently, no discontinuations.

Of interest and in line with previous studies, longer hospitalization and treatment with vancomycin or metronidazole was significantly associated with nonclearing of VREfm (37, 38). Antibiotics that specifically kill anaerobic bacteria, like metronidazole, have been shown to dispose hospitalized patients to density of VREfm colonization (39). One study demonstrated how VREfm came to entirely dominate the intestinal flora in hemopoietic stem cell patients (35). Another study showed that vancomycin depleted most bacteria found in the intestinal tract and that recovery to baseline microbiota is highly dependent on the individual (18). Oral vancomycin, and to a lesser extent metronidazole, has been shown to have a profound effect on the gut microbiome and to drive VREfm colonization (37). Thus, in order to lessen the burden of the VREfm epidemic seen around the globe, antibiotic stewardships and good infection control precautions are still the golden standards.

As for the microbiome analyses, there was no significant difference between the LGG and placebo groups in terms of alpha diversity, nor was there a significant difference between clearers and nonclearers of VREfm. The fact that the LGG and placebo groups differed from one another in terms of beta diversity was most likely not attributable to the ingestion of LGG but could possibly be explained by the placebo group having a higher range in alpha diversity at baseline.

It is not surprising that there is no difference in the alpha diversity between the LGG and placebo groups in our study, as most of our patients were hospitalized and had received broad-spectrum antibiotics at baseline. To restore the gut microbiota to a nondysbiotic state in hospitalized patients who have received broad-spectrum antibiotics, possibly more than one probiotic strain is needed, and thus a cocktail of many different probiotics or a combination of probiotics and prebiotics could be used. Discussions of the benefits of single-strain versus multistrain mixtures are ongoing in the field of probiotics (40). A study of elderly patients receiving a prebiotic mixture did not show any effect on alpha or beta diversity (41). In contrast, case studies using fecal microbial transplants show good effects on VREfm eradication from the gut (42–45).

Contrary to what we would have hypothesized, more clearers than nonclearers had a high relative abundance of *Enterococcus* at baseline. This finding did not persist at week 4, where the relative abundance was lower in clearers than in nonclearers. One could speculate that having a high abundance of E. faecium, which also includes VSEfm (vancomycin-susceptible E. faecium), could help outcompete VREfm. One study looking at VREfm acquisition showed that the difference between those that acquired VREfm and those that did not differed only in the relative abundance of *Enterococcus*, with a higher abundance in those that acquired VREfm but with no difference in the Shannon diversity index (21). It has been suggested that enterococcal expansion could be a biomarker for the microbiota’s susceptibility to colonization by MDR bacteria (46). More research in this area is warranted.

An experimental gap of this study was the limited number of samples. We were able to include only 81 of the planned 162 patients. Still, considering that the natural clearance of VREfm was much higher than anticipated, a higher number of patients might not have led to different results. A strength of this study was the complementing microbiome analyses of the fecal samples, leading to insight into other aspects of VREfm clearance.

We identified a suboptimal detection of Gram-negative bacteria in the positive control, as has been reported by others (47). However, we believe that this has influenced our results only marginally, as Gram-negative bacteria were detected in high relative abundance in many of our samples.

Altogether, our study did not find an effect of LGG in clearing the intestinal colonization of VREfm, nor did it have any effect on the diversity of the microbiome. However, the natural clearance of VREfm was almost 60% after 4 weeks and, thus, much higher than anticipated.

## MATERIALS AND METHODS

### Ethics approval and consent to participate.

The trial has been approved by the Danish Data Protection Agency (AHH-2018-001, 06108), and permission for human experiments and recruitment of participants was obtained from the Scientific Ethics Committee for Copenhagen Regional Hospitals (permission no. H-18011991). As Lactobacillus rhamnosus is considered a dietary supplement and not a pharmaceutical, no authorization by the Danish Medical Agency was required. The study is performed in accordance with the requirements of the Revised Declaration of Helsinki. The study is registered with the title “Probiotic Efficacy in VRE Eradication (PROVE)” at ClinicalTrials.gov (NCT03560700).

### Study overview.

Between May 2018 and November 2020, we conducted a multicenter, double-blinded, randomized, placebo-controlled trial in hospitalized adult patients at three acute-care hospitals in the Capital Region of Denmark.

### Study population.

Adult patients were assessed for eligibility if they were >18 years of age and had a PCR-positive rectal swab for *van*A within the past 7 days. Patients were identified through screening of our laboratory information system. The following exclusion criteria were applied: symptomatic VREfm infection, serious immunodeficiency, pancreatitis, planned or recent intraabdominal operation within a time window of 14 days, parenteral nutrition, antibiotic active against VREfm 1 month prior to inclusion, terminal disease with expected survival time of <3 months, pregnant or lactating women, admission to an intensive care unit, and inability to sign written informed consent. The inclusion process is illustrated in Fig. 1, which is based on the CONSORT flowchart (48).

All patients were included in safety assessment and analyzed for number of VREfm infections (isolated from nonintestinal foci), as well as any LGG invasive infections. At the 4-week interview, patients were specifically asked for adverse effects (AEs) and severe adverse effects (SAEs) related to LGG.

### Randomization and intervention.

Patients were randomized to receive one capsule containing 3 × 10^10^ CFU of LGG (Chr. Hansen A/S, Hoersholm, Denmark) twice daily or a placebo capsule identical in appearance. The randomization list was drawn up for each of the three sites using the SAS proc plan procedure (49). Access to the randomization list was limited to the staff that generated the list. Study participants, staff, and data analysts were blinded; only the study coordinator at Chr. Hansen A/S knew the treatment assignment. Compliance with the study intervention was assessed by counting the remaining capsules at the end of the intervention period (week 4 visit).

### Study outcomes.

The primary outcome was the colonization status of VREfm after the 4-week intervention. During the study period, *van*A was the dominant VREfm clone in Denmark, which is why this study was based on isolates from patients with *van*A VREfm (4). After 4 weeks, a fecal swab was assessed by a *van*A PCR.

Secondary outcomes for a subgroup of patients willing to come in for follow-up visits included clearance of VREfm colonization at weeks 8, 16, and 24, also assessed by *van*A PCR, and changes in fecal microbiome composition at baseline, week 4, and week 24.

**FIG 3:**
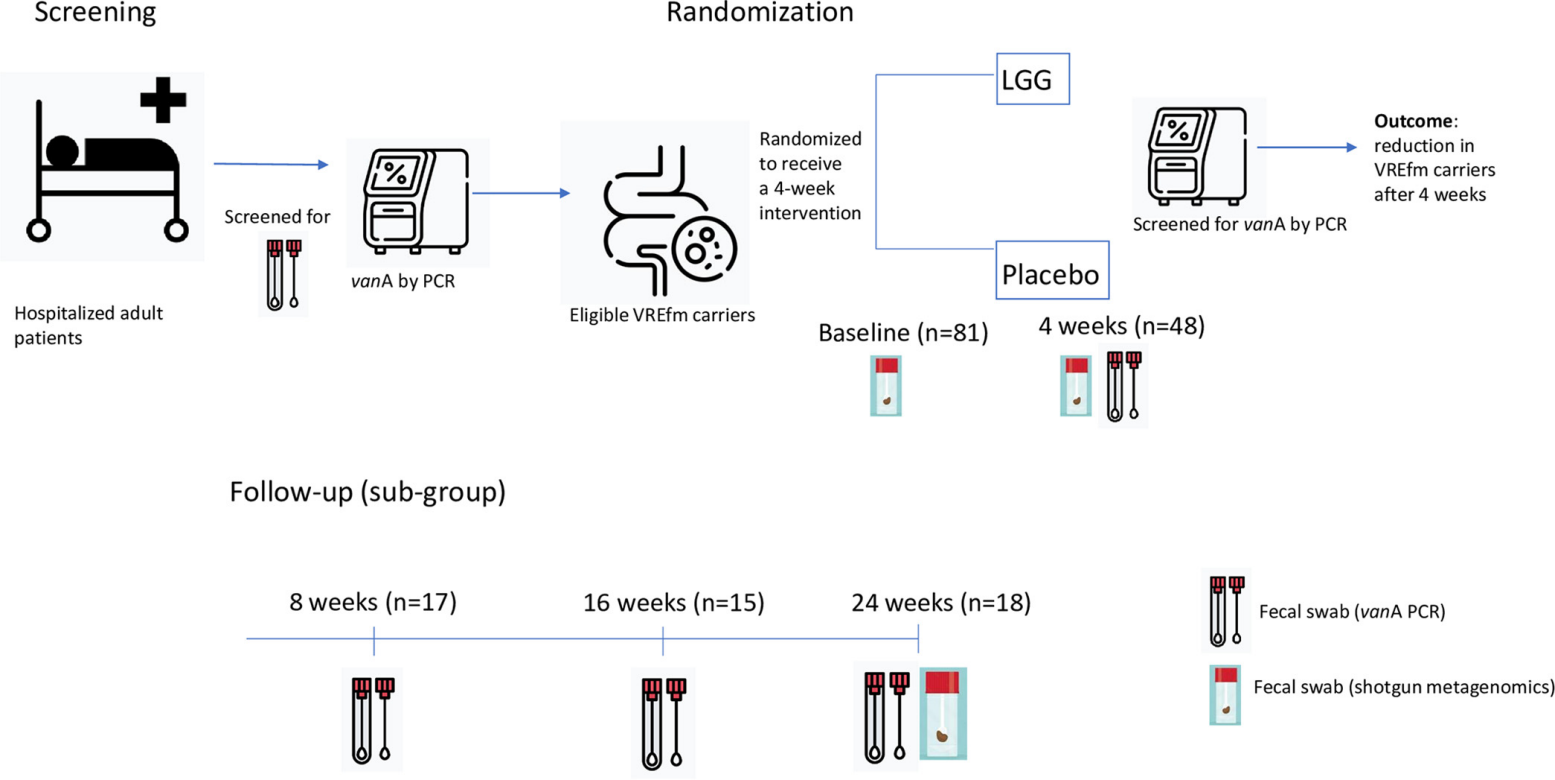
Study set-up for participants analyzed in outcome analyzes and number of patients in follow-up visits at each time point.

For the study set-up, please refer to Fig. 3.

### Sample size calculation.

Based on prior studies, our assumption was that 50% of patients treated with LGG would clear their VREfm by week 4 compared to 25% in the placebo group (26–28). With a power of 80% and significance level of 5%, each group would need 57 patients. Accounting for an expected dropout of 30%, this would give 81 patients in each group.

### Statistical analyses of the clinical data.

As we gave a very high dose of LGG (60 billion CFU daily), study participants were included in analyses if they had ingested a minimum of 50% of the LGG/placebo capsules during the 4-week intervention. All continuous variables are presented as median with interquartile range (IQR). Comparisons between treatment with LGG and placebo were analyzed using Wilcoxon rank-sum test. Categorial variables are presented as count and percentages and odds ratios (OR) with 95% confidence intervals (CI), and comparisons between groups were done using chi-squared or Fisher’s exact test.

Additionally, an analysis comparing clearers versus nonclearers for known risk factors associated with VREfm acquisition was performed (37, 50–52). These risk factors included treatment with one of the following high-risk antibiotics, metronidazole, vancomycin, or cefuroxime, age, Charlson comorbidity score, and length of hospitalization before and after inclusion in the study. We also included piperacillin-tazobactam, as this was part of our antibiotic stewardship during the study period and it was the most frequently used antibiotic. These comparisons were again tested by Wilcoxon rank-sum test, chi-squared test, or Fisher’s exact test. All data management and analyses were performed using R version 4.1.0 (53).

### Fecal samples and sequencing.

Rectal swabs (BD, Fecal Swab, Copan, Italia) were first screened by an in-house *van*A PCR for rapid detection of VREfm. The *van*A-positive samples were incubated overnight in brain heart infusion broth and cultured on VRE ChromID (bioMérieux) and blood agar for confirmation. All analyses took place at the Departments of Clinical Microbiology at Herlev or Hvidovre University Hospitals.

For microbiome analyses, DNA was isolated directly from fecal swab samples (sterile foam tipped applicator, Puritan Medical Products, ME) and processed using ultra-deep microbiome prep (Molzym GmbH & Co, Germany). One extraction control (blank) and one positive control (ZymoBIOMICS Microbial Community Standard, catalog no. D6300) were included. DNA libraries were constructed using the Nextera XT DNA sample preparation kit (Illumina, Denmark) according to the manufacturer’s protocol and sequenced on the Nextseq 500 (Illumina Inc., San Diego, USA) using 2 by 150 bp paired-end reads. All samples including the controls were run together on one NextSeq run.

### Bioinformatic analysis of the microbiome data.

Fastq read files were trimmed using fastp v. 0.20.1 (54) with –qualified_quality_phred of 20 and minimum read length of 50. FastQC v. 0.11.8 was used to assess the quality of the reads before and after trimming (55). The data were depleted of human sequences by aligning the trimmed reads to the human genome (hg38, University of California, Santa Cruz) using bowtie2 v. 2.3.4.1 with end-to-end alignment and maximum fragment length for valid paired-end alignments (-X) of 2000 (56). Clade-based microbial profiling of the human depleted reads was performed with MetaPhlAn3 v. 3.0.10 with addition of the parameters –ignore_eukaryotes and -t rel_ab_w_read_stats (57). Relative abundance tables for all samples were merged using the MetaPhlAn3 script merge_metaphlan_tables.py.

### Taxonomic and diversity analyses.

The taxonomic data were processed in R version 4.1.0 (53). Rare taxa with sample-level relative abundances of <0.01% and taxa found in only one sample were filtered out. For presentation in bar plots, families present at relative abundance of <1% were grouped as “Other.” The 10 most abundant genera were selected based on highest mean relative abundance, and the top 10 genera were selected for VREfm clearers and nonclearers separately for both baseline and week 4 samples.

Species richness and Shannon diversity were calculated using the vegan package (58), and differences were assessed with paired Wilcoxon signed rank test. ggplot2 was used for visualization of richness and Shannon diversity (59). Beta diversity analysis was performed using QIIME2 v. 2021.2 (60) using the species level estimated read counts generated by MetaPhlAn3. Read count values representing below 0.01% relative abundance and species present in only one sample were filtered out using the QIIME2 feature-table plugin. Aitchison distance was used as beta diversity metric to account for the compositionality of the data (32, 61) and was calculated using the QIIME2 diversity plugin adding a pseudocount of 1. Differences in beta diversity were assessed based on Aitchison distances with PERMANOVA. Principal-coordinate analysis (PCoA) was calculated using the diversity plugin and visualized using the emperor plugin (62).

Differential abundance analysis was performed using the R package ALDEx2 (63). The data were transformed using the aldex.clr function, which generates random instances of the centered log-ratio-transformed values, with a model matrix representing the groups, mc.samples of 1,000 (number of Monte-Carlo instances), and denom of “all” (using all features as denominator for the geometric mean calculation). The function aldex.glm (implementing a generalized linear model) was used to generate test statistics for the output of aldex.clr. *P* values were adjusted using the Benjamini-Hochberg procedure. Effect sizes and differences between the groups were calculated using aldex.glm.effect. An effect size of 1 was used as cutoff to denote differentially abundant features, as recommended by the package developers. The following comparisons were made: placebo baseline versus placebo week 4, placebo baseline versus LGG baseline, LGG baseline versus LGG week 4, and placebo week 4 versus LGG week 4.

### Data availability.

Raw sequencing reads depleted of human reads are deposited in NCBI Sequence Read Archive under BioProject no. PRJNA777429.
