# Supplementary material for: No Effect of Lactobacillus rhamnosus GG on Eradication of Colonization by Vancomycin-Resistant Enterococcus faecium or Microbiome Diversity in Hospitalized Adult Patients
Source: Microbiol Spectr. 2022 Apr 27;10(3):e02348-21. doi: 10.1128/spectrum.02348-21 (PMC9241610; doi:10.1128/spectrum.02348-21)
Supplement: SUPPLEMENTAL FILE 1 — Supplemental material. Download spectrum.02348-21-s001.pdf, PDF file, 0.4 MB [file spectrum.02348-21-s001.pdf]

Supplementary Figure 1.

Fecal microbiome composition at family level. All taxa with a relative abundance of less than 1% are grouped together as “Other”.
Pt: patient, B: baseline, W4: week four, W24: week 24

Supplementary figure 2

Relative abundance of *E. faecium* at species level. Bars are colored according to which patients had cleared VREfm at week four, where green represents clearers and blue represents non-clearers.

Supplementary Figure 3.

The relative abundance of the 10 most abundant genera at baseline and four weeks for the LGG and placebo groups (A) and VREfm clearers and non-clearers (B).

Supplementary Figure 4.

Alpha diversity in terms of richness (left) and Shannon diversity (right) for VREfm clearers vs. VREfm non-clearers.

Supplementary table 1

P-values, Benjamini-Hochberg (BH) adjusted p-values, and effect sizes from ALDEx2 analysis of differentially abundant species. Only associations with P-values <0.05 are shown. *Lactobacillus rhamnosus*, the only species having either adjusted p-value <0.05 or effect size >1 is highlighted in green.

Supplementary table 2

Supplementary table 2. Relative abundance of species detected in the positive control ZymoBIOMICS Microbial Community Standard.

Supplementary data

Merged abundance table of relative abundances from MetaPhlan3 including all taxonomic levels (first sheet “MetaPhlan3 relative abundance”), merged abundance table of estimated read counts from MetaPhlan3 including all taxonomic levels (second sheet “MetaPhlan3 read counts”), and filtered species level relative abundances from MetaPhlan3 with abundances <0.01% removed and species found in <2 samples removed (third sheet “Species relative abundances”).

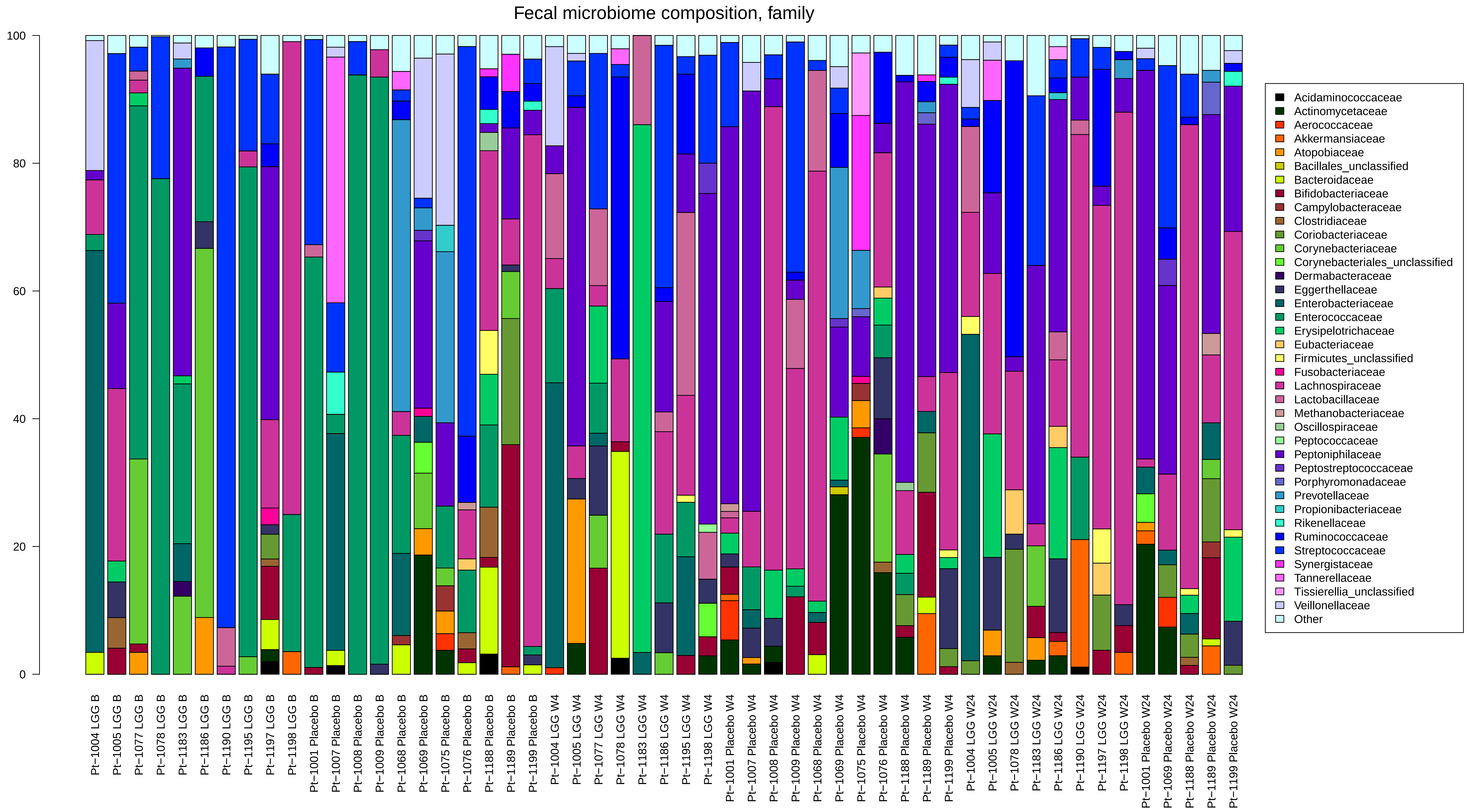

Fecal microbiome composition, family
100
80
60
40
20
0
Acidaminococcaceae
Actinomycetaceae
Aerococcaceae
Akkermansiaceae
Atopobiaceae
Bacillales_unclassified
Bacteroidaceae
Bifidobacteriaceae
Campylobacteraceae
Clostridiaceae
Coriobacteriaceae
Corynebacteriaceae
Corynebacteriales_unclassified
Dermabacteraceae
Eggerthellaceae
Enterobacteriaceae
Enterococcaceae
Erysipelotrichaceae
Eubacteriaceae
Firmicutes_unclassified
Fusobacteriaceae
Lachnospiraceae
Lactobacillaceae
Methanobacteriaceae
Oscillospiraceae
Peptococcaceae
Peptoniphilaceae
Peptostreptococcaceae
Porphyromonadaceae
Prevotellaceae
Propionibacteriaceae
Rikenellaceae
Ruminococcaceae
Streptococcaceae
Synergistaceae
Tannerellaceae
Tissierellia_unclassified
Veillonellaceae
Other
Pt−1004 LGG B
Pt−1005 LGG B
Pt−1077 LGG B
Pt−1078 LGG B
Pt−1183 LGG B
Pt−1186 LGG B
Pt−1190 LGG B
Pt−1195 LGG B
Pt−1197 LGG B
Pt−1198 LGG B
Pt−1001 Placebo B
Pt−1007 Placebo B
Pt−1008 Placebo B
Pt−1009 Placebo B
Pt−1068 Placebo B
Pt−1069 Placebo B
Pt−1075 Placebo B
Pt−1076 Placebo B
Pt−1188 Placebo B
Pt−1189 Placebo B
Pt−1199 Placebo B
Pt−1004 LGG W4
Pt−1005 LGG W4
Pt−1077 LGG W4
Pt−1078 LGG W4
Pt−1183 LGG W4
Pt−1186 LGG W4
Pt−1195 LGG W4
Pt−1198 LGG W4
Pt−1001 Placebo W4
Pt−1007 Placebo W4
Pt−1008 Placebo W4
Pt−1009 Placebo W4
Pt−1068 Placebo W4
Pt−1069 Placebo W4
Pt−1075 Placebo W4
Pt−1076 Placebo W4
Pt−1188 Placebo W4
Pt−1189 Placebo W4
Pt−1199 Placebo W4
Pt−1004 LGG W24
Pt−1005 LGG W24
Pt−1078 LGG W24
Pt−1183 LGG W24
Pt−1186 LGG W24
Pt−1190 LGG W24
Pt−1197 LGG W24
Pt−1198 LGG W24
Pt−1001 Placebo W24
Pt−1069 Placebo W24
Pt−1188 Placebo W24
Pt−1189 Placebo W24
Pt−1199 Placebo W24

Enterococcus faecium relative abundance

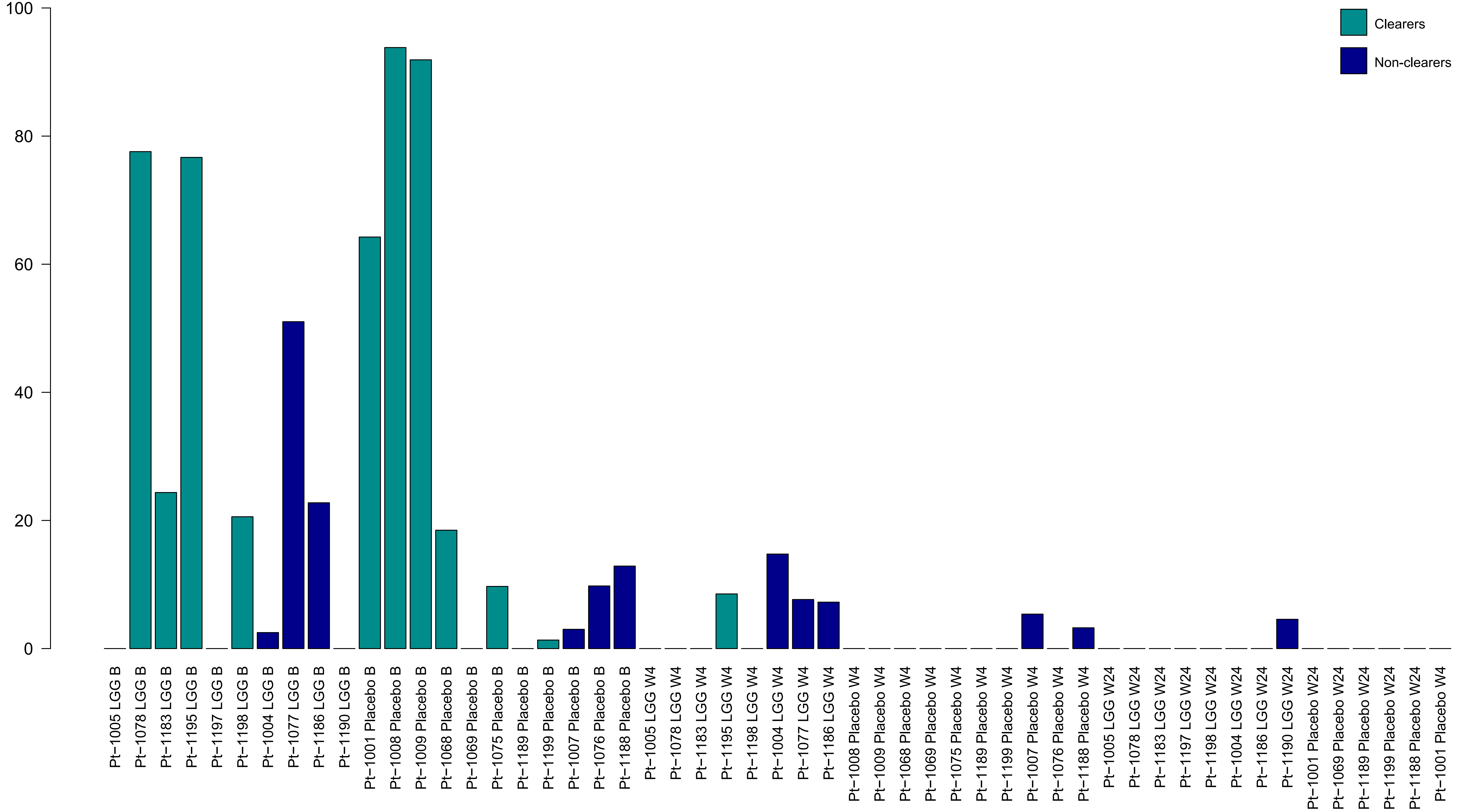

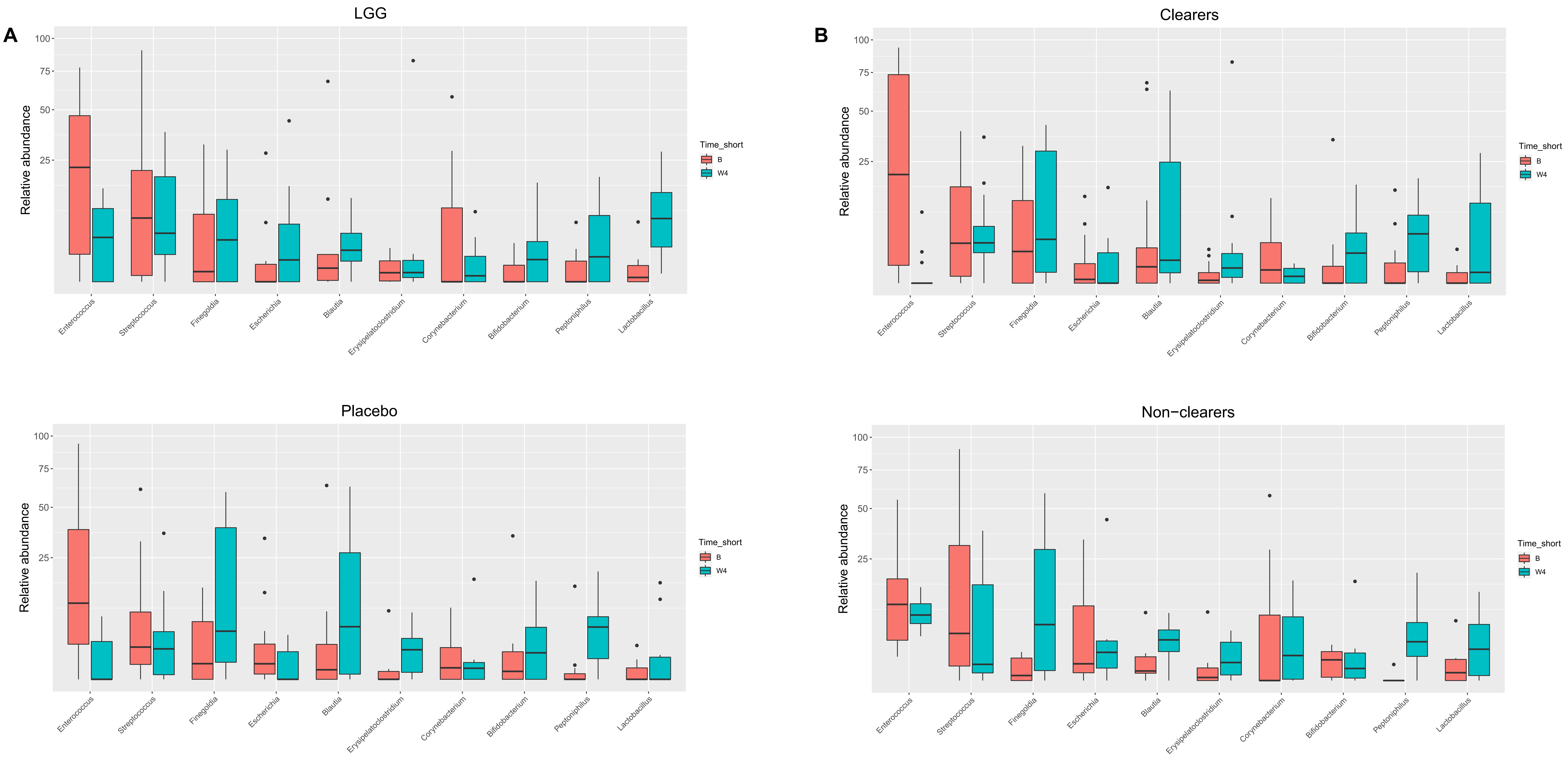

A
LGG
B
Clearers
Placebo
Non−clearers
Relative abundance
100
75
50
25
Time_short
B
W4
Enterococcus
Streptococcus
Finegoldia
Escherichia
Blautia
Erysipelatoclostridium
Corynebacterium
Bifidobacterium
Peptoniphilus
Lactobacillus

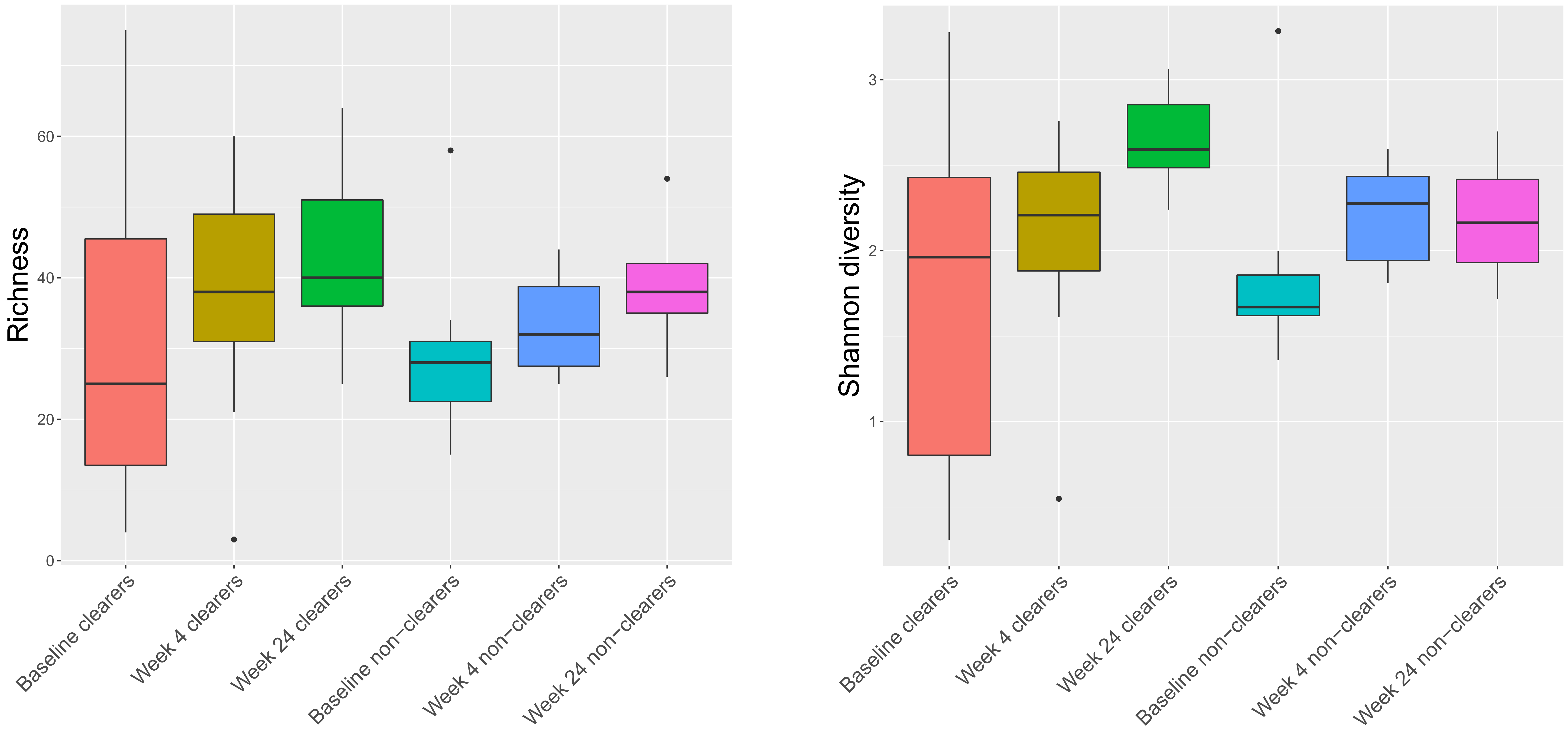
Richness
0
20
40
60
Baseline clearers
Week 4 clearers
Week 24 clearers
Baseline non−clearers
Week 4 non−clearers
Week 24 non−clearers
Shannon diversity
1
2
3
Baseline clearers
Week 4 clearers
Week 24 clearers
Baseline non−clearers
Week 4 non−clearers
Week 24 non−clearers

Supplementary table 1. P-values, Benjamini-Hochberg (BH) adjusted p-values, and effect sizes from ALDEx2 analysis of differentially abundant species. Only associations with P-values <0.05 are shown. *Lactobacillus rhamnosus* , the only species having either adjusted p-value <0.05 or effect size >1 is highlighted in green.

	Placebo week four vs. placebo baseline			LGG baseline vs. placebo baseline			LGG week four vs. LGG baseline			LGG week four vs. placebo week four		
	**p-value**	**p-value (BH adjusted)**	**Effect size**	**p-value**	**p-value (BH adjusted)**	**Effect size**	**p-value**	**p-value (BH adjusted)**	**Effect size**	**p-value**	**p-value (BH adjusted)**	**Effect size**
Anaerococcus_vaginalis	0,023404113	0,971942336	0,10600932				0,0136538	0,912236975	0,229249998			
Bacteroides_thetaiotaomicron				0,03678445	0,969368855	-0,158673731						
Clostridium_bolteae	0,033458213	0,929346553	-0,165866103									
Clostridium_innocuum	0,028048765	0,999401477	0,564029486									
Corynebacterium_pyruviciproducens				0,034440156	0,98991611	0,257240511				0,006013984	0,716066857	0,692519781
Eisenbergiella_massiliensis							0,025495575	0,999482973	0,771001845	0,010278796	0,970600934	0,772511707
Enterococcus_faecium	0,002208373	0,502384499	-0,402519408									
Lactobacillus_rhamnosus							0,000854587	0,203843976	1,603639695	6,79E-05	0,016806376	1,595717923
Staphylococcus_haemolyticus	0,016047214	0,813218397	-0,238691377									
Varibaculum_cambriense	0,037175765	0,999195682	0,571595745									

Supplementary table 2. Relative abundance of species detected in the positive control ZymoBIOMICS Microbial Community Standard.

**Species**	**Relative abundance**
Listeria_monocytogenes	1.916.108
Bacillus_intestinalis	103.903
Escherichia_coli	0.02298
Salmonella_enterica	0.01434
Lactobacillus_fermentum	2.182.715
Enterococcus_faecalis	2.742.585
Staphylococcus_aureus	2.084.013
Staphylococcus_argenteus	0.31818
